# Toward sustainable space exploration: a roadmap for harnessing the power of microorganisms

**DOI:** 10.1038/s41467-023-37070-2

**Published:** 2023-03-21

**Authors:** Rosa Santomartino, Nils J. H. Averesch, Marufa Bhuiyan, Charles S. Cockell, Jesse Colangelo, Yosephine Gumulya, Benjamin Lehner, Ivanna Lopez-Ayala, Sean McMahon, Anurup Mohanty, Sergio R. Santa Maria, Camilla Urbaniak, Rik Volger, Jiseon Yang, Luis Zea

**Affiliations:** 1 UK Centre for Astrobiology, School of Physics and Astronomy, University of Edinburgh, Edinburgh, UK; 2 Department of Civil & Environmental Engineering, Stanford University, Stanford, CA USA; 3 Center for Utilization of Biological Engineering in Space, Berkeley, CA USA; 4 Everest Innovation Lab, Honolulu, HI USA; 5 Cemvita Factory, Westminster, CO USA; 6 Centre for Microbiome Research, Queensland University of Technology, Brisbane, QLD Australia; 7 DMEC, The Haag, Netherlands; 8 Universidad del Valle de Guatemala, Guatemala City, Guatemala; 9 Blue Marble Space Institute of Science, 600 1st Ave, Floor 1, Seattle, WA 98104 USA; 10 Department of Biotechnology, SRM Institute of Science and Technology, Kattankulathur, India; 11 Space Biosciences, NASA Ames Research Center, Mountain View, CA USA; 12 KBR, Moffett Field, Mountain View, CA USA; 13 NASA Jet Propulsion Laboratory, California Institute of Technology, Pasadena, CA USA; 14 ZIN Technologies Inc, Middleburg Heights, OH USA; 15 Department of Biotechnology, Delft University of Technology, Delft, The Netherlands; 16 Biodesign Center for Fundamental and Applied Microbiomics, Biodesign Institute, Arizona State University, Tempe, AZ USA; 17 BioServe Space Technologies, University of Colorado Boulder, Boulder, CO USA

**Keywords:** Biotechnology, Applied microbiology, Scientific community, Element cycles

## Abstract

Finding sustainable approaches to achieve independence from terrestrial resources is of pivotal importance for the future of space exploration. This is relevant not only to establish viable space exploration beyond low Earth–orbit, but also for ethical considerations associated with the generation of space waste and the preservation of extra-terrestrial environments. Here we propose and highlight a series of microbial biotechnologies uniquely suited to establish sustainable processes for in situ resource utilization and loop-closure. Microbial biotechnologies research and development for space sustainability will be translatable to Earth applications, tackling terrestrial environmental issues, thereby supporting the United Nations Sustainable Development Goals.

## Abstract (web summary)

Establishing sustainable approaches for human space exploration is key to achieve independency from terrestrial resources, as well as for ethical considerations. Here the authors highlight microbial biotechnologies that will support sustainable processes for space-based in situ resource utilization and loop-closure, and may be translatable to Earth applications.

## Introduction

Humanity may be tempted to view the cosmos as a rich reservoir of infinite resources. However, the practical reality of space exploration tells a different story. The need for sustainability in space exploration, as well as the exploitation of space, is becoming more evident with the strengthening desire for the expansion of human activities beyond Earth-orbit, pursued by public and private sectors alike. When applied to space, the concept of sustainability has often been understood as "ensuring that all humanity can continue to use outer space for peaceful purposes and socioeconomic benefit now and in the long term"^1^. Until now, it mainly referred to the need to control, regulate, and remove space debris from low Earth orbit (LEO)^2,3^ and planetary protection (which promotes the implementation and development of the responsible exploration of the solar system, in order to protect the space environments and Earth)^3^. As humans aspire to venture into deep space, the definition of this concept shifts and expands, and the self-sustainability of mission operations becomes a critical aspect. Loop-closure, which indicates the recycling and the reuse of resources toward the establishment of a circular economy, could greatly enhance the sustainability of space exploration, and it is key not only to minimise the costs of resupply of resources from Earth but also for ethical considerations associated with space waste generation and the preservation of extra-terrestrial environments^4–10^. The United Nations resolved that outer space activities should minimise impacts on the space environment, as well as on Earth, taking into account the 2030 agenda for Sustainable Development^11,12^.

The biggest impediment to progress on this frontier is the lack of deployable technologies enabling outposts, extended missions and, in the future, settlements, to sustain themselves through in situ resource utilization (ISRU) and maximised recycling of resources^5^. In addition to mechanical/physical/chemical approaches, biotechnologies broadly and microorganisms specifically will help enable long-term life-support and habitat systems’ performance (loop-closure), as well as ISRU, manufacturing and energy collection/storage^6,7,13–17^. Microbiological approaches can be self-sustaining with occasional monitoring and maintenance, owing to their resilience, and could overall require less energy than physicochemical approaches^16^.

Here, we focus on the pivotal roles that microorganisms can play in the development of technologies for sustainable human exploration of deep space, considering two main aspects: (i) the requirement for mature biotechnologies and bioprocesses to allow near closed-loop operations of mission functions, such as life-support, to increase autonomy and sustainability; and (ii) the need to reduce supply chain dependency for the expansion of human presence in space. The approaches presented here are based on processes and technologies currently implemented on Earth at different technology readiness levels (TRL), which must be adapted to meet the specific requirements and challenges of the space environment. Selecting the most suitable bioprocess and most applicable microorganism for any given space application is non-trivial, as terrestrial technologies are rarely readily adaptable to the harsh conditions of space^18^. Therefore, extensive research and development are compulsory to increase TRL to the point where these technologies can be successfully implemented in space^16^. Finally, microbial biotechnologies aimed to increase the sustainability of space exploration may be translatable to Earth applications for advancement towards a circular economy, further supporting the United Nations Sustainable Development Goals (SDGs)^11,12^.

## Habitat air bioremediation

**Fig. 1:**
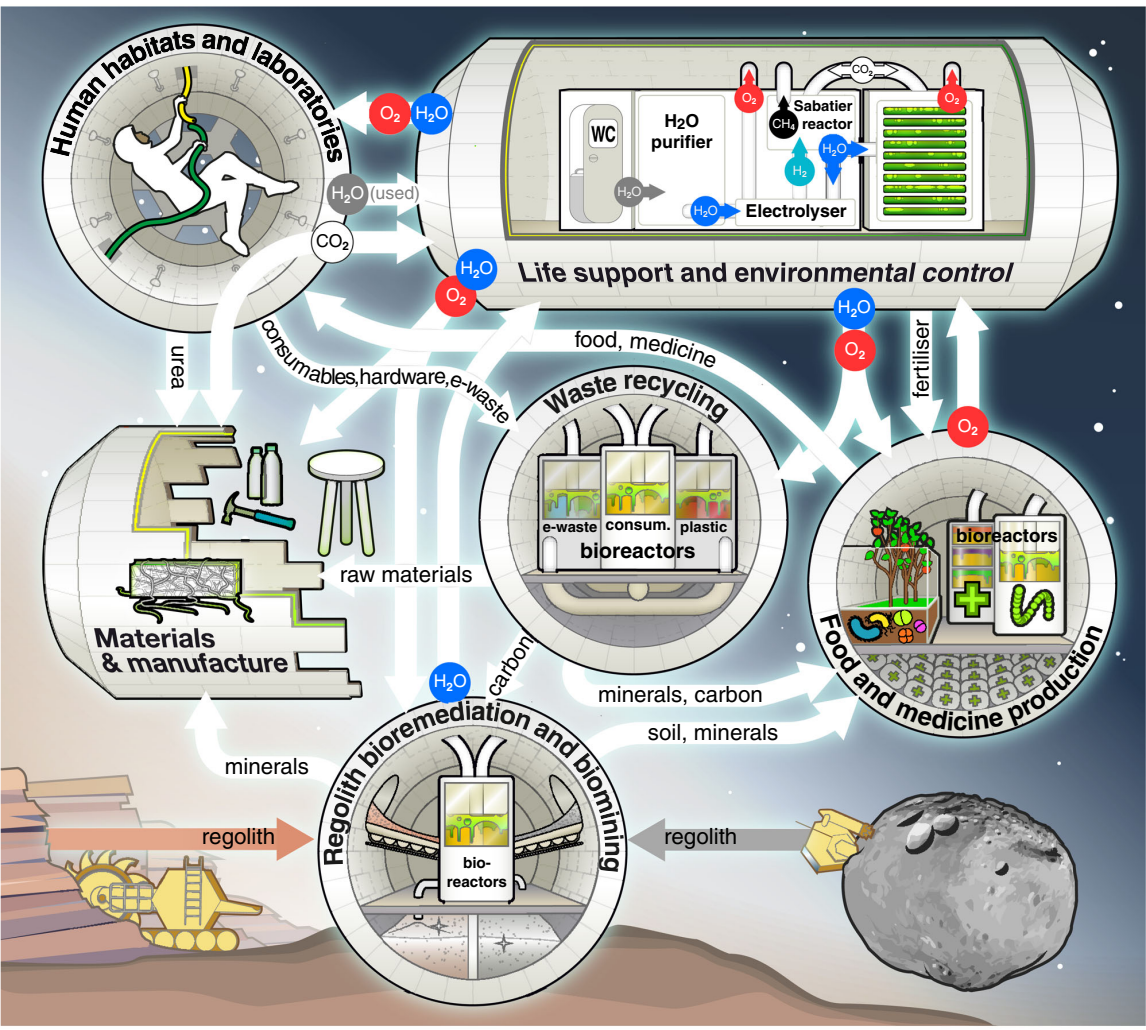
Graphical rendering of a microbial biotechnology-based life-support system in an agnostic space environment. Microorganisms are included in all the functional compartments, including the ‘Human habitats and laboratories’ (i.e., human microbiome) and those that also require mechanochemical reactors. Up- and recycling of different resource streams and loop-closure is indicated by white arrows, supported by in situ resource utilization (coloured arrows), as applicable.

A spacecraft’s Environmental Control and Life Support System (ECLSS) manages water supply, atmospheric pressure and composition (O_2_, CO_2_, and inert-gas levels), temperature and relative humidity, as appropriate for human operations and conducive to comfortable living^19^. Carbon dioxide on the International Space Station (ISS) is currently scrubbed from the cabin air and converted in a Sabatier reaction to recover oxygen, while the by-product (methane) is vented into space. This has two drawbacks: (i) hydrogen is required as a substrate, production of which is energy intense; (ii) hydrogen and carbon are lost, requiring constant resupply. Recycling CO_2_ and/or methane back into the organic matter could improve loop-closure and preserve resources. Microbial bioremediation can support the removal of CO_2_ from the atmosphere in habitats, while also supplementing the generation of breathable oxygen^20^. The captured carbon may be upcycled again and reintegrated into the resource cycle, e.g., for the biomanufacturing of food/supplements. Alternatively, captured carbon may be sequestered for end-use (Fig. 1), e.g., for bioconcrete production or microbial-electrolytic carbon capture, as described later. Direct air capture of CO_2_ could reduce the need for energy-intensive auxiliary processes and shorten the recycling loop^21^. Researchers at the Center of Applied Space Technology and Microgravity (ZARM, University of Bremen, Germany) are exploring the application of cyanobacteria like *Anabaena* sp. PCC 7938 in a Martian context, taking advantage of its carbon- (photosynthetic) and nitrogen-fixing (diazotrophic) capabilities. Low carbon concentration in habitats or spacecraft yields a conversion efficiency two- to five-fold lower than with preconcentrated CO_2_^22^. Hence, the most applicable approach will strongly depend on the mission scenario, in terms of size, extent and duration. Reversible carbon scrubbers^23^ combined with microbial conversion may enable a sustainable and near-closed process for deep space travel and exploration missions or initial human settlements^24,25^.

## Human waste processing and reclamation

Human waste management poses a challenge for human space exploration, and proposed solutions commonly focus on how to compact, sterilise and dispose of human waste, rather than recycling it^26^. On the ISS, human waste is currently stabilised, dried and ejected from the station to burn up in Earth’s atmosphere^27^. This method is clearly not sustainable for long-distance missions, as it represents a sink of potentially useful resources. Solid human waste could instead be used as a feedstock for production of food and edible supplements, as well as nutrients/fertiliser for microbial or plant-based Life Support System (LSS) components (Fig. 1). Many physicochemical approaches to solid waste management, such as pyrolysis or incineration, are being proposed^26^. However, microbiologically supported processes could enhance human waste recycling by enhancing loop-closure. The European Space Agency (ESA) supported MELiSSA project (Micro-Ecological Life Support System Alternative) includes a series of interconnected bioreactors for LSS; it is a good example of how diverse types of waste-streams, including human metabolic end-products, could be upcycled using microorganisms^5^. Although anaerobic waste processing is usually considered less time-efficient than aerobic methods, it can achieve comparable degradation rates as physicochemical processes. A two-component system, which anaerobically converts liquid and solid human waste to protein- and lipid-rich microbial biomass for food production, has been developed^28^.

## Food production

The ability to produce food in space will be of paramount importance to achieve sustainability and self-sufficiency on long-duration space missions and may be significantly enhanced by microbial biotechnology. For example, the plant microbiome will play a pivotal role in the success of crop production in space. As with the human microbiome, the plant-associated microbial community provides a multitude of benefits to the host, such as promoting plant growth, stimulating phytohormone production, controlling pathogens, regulating immune function, and alleviating abiotic and biotic stressors^29^. Symbiotic bacteria are also essential in solubilizing nutrients from the environment and converting them into bioavailable forms, and improving soil fertility^29,30^. Nitrogen recovery for food production can, for instance, be accomplished by microbial-mediated nitrogen fixation from the atmosphere, or nitrification of urine, mediated by synthetic microbial communities (see section ‘Human waste processing and reclamation’)^31^.

For long-duration space travel, the use of agricultural probiotics should be considered to help grow robust, stable, and healthy crops in space. Agricultural probiotics are already used in farming on Earth as plant biostimulants^32^, as biofertilisers^33^ and for bioremediation to improve soil quality^34^. A better understanding of how the composition and function of the plant microbiome changes in spaceflight can help to customise probiotic supplements for space farming. For example, the concept of rhizobial inoculants is already in practice: seeds are inoculated with chosen strains of bacteria and fungi that can improve crop productivity and environmental sustainability^35^. Additional attributes of the inoculum geared towards spaceflight could also consist of bacteria/fungi that help maximise resource efficiency, thereby requiring less input for optimal growth. Algae and cyanobacteria may be cultivated not only for air revitalization, as discussed before, but also as a food source and to support plant growth^9,20^.

For successful human outposts on the Moon or Mars, crews will have to produce their own food, taking advantage of local (in situ) resources, like regolith (see the section ‘Soil remediation’). Agricultural methods may include soil-based farming, hydroponics, cellular agriculture, etc. Creation of a greenhouse-like infrastructure, by the example of the ISS’ Advanced Plant Habitat^36^, and sufficient light, water supply, soil nutrients, and other necessary parameters for crop growth may enhance loop-closure. These and other resources could be obtained from other (biologically supported) LSS and ISRU compartments (Fig. 1).

## Pharmaceutical synthesis

The idea of in situ manufacturing of drugs in space and taking advantage of microgravity has been around for decades^37^. The slower growth of crystals in the microgravity environment has enabled the discovery of new compounds^38,39^. The altered expression of microbial virulence and/or pathogenesis-related genes in space can elucidate the underlying mechanisms and assist with identifying new vaccines and treatments^39,40^. The space environment also brings physiological changes to astronauts during space flight^39^, providing accelerated models for aging and disease in drug discovery^41^. In the context of human exploration to the Moon or Mars, having a pharmaceutical foundry in space can remove the dependency on a supply of medicines from Earth and provide ways to manufacture drugs on demand^39^. Many of the carry-along pharmaceuticals will expire during a Mars-return mission of standard duration (1.5 or 2.7 years), or have reduced potency due to exposure to the space environment^42^, which tends to alter the active pharmaceutical ingredients and increases the concentrations of degradants or impurities^42^. To date, no studies have been reported on the stability of biological drugs such as monoclonal antibodies, versatile therapeutics capable of treating various space-relevant diseases, including osteoporosis, infections and inflammations.

Microbes have long been used on Earth for the production of pharmaceuticals and high-value chemicals. Given the extreme conditions and limited resources in space environments, spore-forming bacteria (resisting radiation and/or desiccation), extremophiles and extremotolerants (resisting harsh environments), and photoautotrophs (surviving on inorganic carbon, e.g., cyanobacteria) have been sent to space to investigate their potential for application in biomanufacturing. Where necessary, microbes may also be engineered for the production of pharmaceuticals in space, which is not a trivial task, given the often complex biosynthetic routes to compounds of interest, as well as the diversity thereof. This also impacts downstream processing, given the stringent requirements for, e.g., purity, and because a one-fits-all solution rarely exists^43^. Computational and synthetic biology tools have been used to simplify the complicated metabolic pathways of conventional biotechnological drug production. For instance, acetaminophen, a versatile drug to treat infection and pain, was proposed to be synthesised by extending the shikimate (also known as chorismate) pathway of the cyanobacterium *Synechocystis* sp. PCC 6803^44^. Microbial recombinant protein expression may also be affected in space environments^45^. Some studies showed that protein production in *Pichia pastoris*, *Escherichia coli*, or *Saccharomyces cerevisiae* was higher in simulated microgravity than in Earth-gravity^45^.

Given that the upcoming exploration missions will go beyond LEO, studies of space pharmaceutical synthesis are mainly focused on compounds or nutrients for countering microgravity-induced osteopenia and acute radiation syndrome^43^. Furthermore, there are efforts to investigate ways to compensate for the weakened immune system of astronauts during long-duration space missions, which can be addressed by the consumption of probiotics or prebiotics to maintain a healthy gut microbiome^39,46^.

A source of microbes that is becoming increasingly relevant for pharmaceutical biosynthesis is the human gut microbiome. A recent metagenomic analysis from the Human Microbiome Project showed that human-associated bacteria encode the biosynthetic machinery to synthesise a vast array of secondary metabolites, such as small bioactive molecules (e.g. antibiotics). Many studies have investigated the changes in the composition and functionality of the gut microbiome during spaceflight and in analogue studies^47^. The use of curated microbial communities (e.g. those that consist of non-pathogenic strains) could be considered to reduce the risk of infectious diseases^48^.

## Biomining

Beyond LEO, the cost of the development of infrastructure increases dramatically. Therefore, a cost-effective supply of resources is required. One option is to harness in situ resources available at the destination through biomining^44,49–51^. Biomining, the use of microorganisms to extract valuable metals from crude minerals (e.g., regolith) and mine waste^52^, has seen extensive effort for adaptation to space applications^50,51,53–56^. Space biomining is primarily limited by economics: biologically mediated processes are often less expensive and less environmentally harmful than hydrometallurgical processes but can rarely provide the same high rates of separation. However, when taking into account the environmental and health costs associated with mining activities on Earth, biomining is already cost-effective for a variety of resources (copper, gold, nickel and cobalt extraction are all performed at a commercial scale)^57^.

Chemolithoautotrophs (e.g., iron and/or sulphur-oxidizing microorganisms) could be suitable for biomining of sulphide minerals, occurring in a variety of settings on Mars or in asteroidal materials^58^. Microorganisms with other nutritional preferences (e.g., organotrophs), consortia, or bioengineering techniques could be used elsewhere: the Lunar surface is mainly composed of silica-saturated rocks, with a generally low sulphur content^50,54^. The need for organic nutrients to feed organotrophic microorganisms, which is limited by the availability of carbon in space environments, could be at least partially satisfied in a closed-loop system (Fig. 1).

Microbial extraction of rare earth elements and vanadium from basalt^54,55,58^, and platinum group elements from L-chondrite meteoritic material has been demonstrated on the ISS^55,56,59^. However, further investigations are required to demonstrate the scalability of these systems, improve performance, and adapt the technology to other elements of interest (e.g., silicon, iron, aluminium). Water, oxygen, hydrogen as well as other critical molecules and elements could also be obtained via biomining^50^. These are essential compounds not only from the biotechnological perspective but indeed for any human activity. Knowledge of the ability to perform biomining under Lunar and Martian conditions is scarce or of theoretical character^50,51^ and efforts toward advancing bioleaching capability from extra-terrestrial regolith under space conditions, and toward the development of bioengineering and synthetic biology approaches, are critical to mature technologies.

While prospecting is inherently not sustainable in the long run because of the inevitable depletion of resources (i.e., the ores, in the case of mining), biomining is generally considered more environmentally friendly than traditional mining because it avoids the use of toxic inorganic reagents. This also matters for extra-terrestrial applications, as it could reduce the need of auxiliary resources. It has been proposed that a biomining reactor could be added to a bioregenerative LSS^50^, hence contributing to loop-closure and ISRU (Fig. 1).

## Structural biomanufacturing: bioconcrete, myco-architecture

Construction and maintenance of infrastructure beyond Earth is a necessary yet daunting task considering the scarce resource availability. Conventional methods of construction utilise large amounts of raw materials and require steady maintenance. To make construction and infrastructure maintenance more sustainable, prioritizing repair over a replacement will be essential and can be facilitated by biological self-healing materials.

Concrete and cement are essential on Earth for construction and binding materials. While likely not being used to the same extent in space due to mass constraints, surface structures may to some degree require the binding of minerals for construction. Microbially induced calcite precipitation (MICP), for example, is a biogeochemical process whereby microorganisms precipitate calcium carbonate (CaCO_3_)^60^, which can be used as a binding agent^61^. Additionally, MICP can also help in the bioremediation of toxic compounds and CO_2_ sequestration^62,63^. Microbial-electrolytic carbon capture could be utilised to sequester CO_2_, and it has been estimated that in the United States, 68 million tons of CO_2_ per year could be captured by this process^64^.

Impact events pulverise regolith on planetary surfaces over time, forming extremely fine dust that can hinder equipment function and cause harm to humans^65^. Bacteria with MICP capability could be used to bind and harden regolith, consolidating the dust^66^. Similarly, cyanobacterial biofilms may be useful to control and bind fine regolith^67^. Byproducts of urea degradation-based MICP, such as ammonium and nitrate, could be further upcycled into a closed-loop system (Fig. 1). However, when the release of toxic byproducts must be avoided^68^, alternative microorganisms for calcium carbonate precipitation can be used (e.g., the methane-oxidizing bacterium *Methylocystis parvus*)^69^.

Fungi-based biotechnologies may provide another opportunity to produce sturdy and resistant structures for space applications. Myco-architecture refers to the production of rigid structural components and surfaces using fungi. Fungal mycelia can form dense networks that combine with other materials, like e.g. regolith, to form mycelium-based composites. These are commonly used in various industries on Earth^70^. The National Aeronautics and Space Administration (NASA) has explored this solution for space exploration, by utilizing mycelia as a means to construct rugged furniture and habitat shells for the Martian and Lunar surfaces^71^. While research for space applications is in its infancy, the extraordinary resilience of fungi has even been a subject of investigation for radioprotection^72^. This potentially provides for in situ manufacturing of robust and self-regenerating structures, and engineered living materials, thus avoiding the need for excessive re-supply, and improving the sustainability of space exploration by mitigating the supply chain from Earth.

## Biological collection and storage of energy

Energy collection and storage (e.g., of fuels) is a critical challenge in any remote environment and is particularly dependent on supply chain consistency. Certain anaerobic bacteria (so-called ‘electricigens’, e.g., Desulfuromonas, Geobacter, etc.) can reduce organic waste to generate electric current. Specifically, microbial fuel cells (MFC) utilise microbes to convert chemically bound energy into electricity^73^. Harnessing the reducing power of organic waste to generate electricity, MFC could be coupled with in situ flow-through waste remediation systems^44^. Such microbial systems can be categorised into dark-fermentation and photobiological processes, and they can use wet biomass from, for example, waste streams^74,75^. In a similar fashion, and quasi-opposed to MFC, microbial electrosynthesis (MES) can be used to convert electricity (back) into chemical compounds for e.g., energy storage, to bridge intermittent availability and demand^76^. Some evidence also indicates the possibility of generating hydrogen through nanoparticle production from Lunar regolith^77,78^. Hydrogen, methane, and other biofuels can also be produced from other in situ resources, such as water and inorganic carbon, photo- or lithoautotrophically (e.g., with cyanobacteria and various algae species), to generate stable energy carriers^6,78^. These could be liquid compounds with high energy density, such as butanediol, as means for storage of energy, to supplement or complement chemically derived bipropellants (hydrogen/methane and oxygen)^76,78^. In many cases, using electricigens, hydrogenic, methanogenic, and biofuel-producing microorganisms, waste products and in situ resources can be upcycled for conversion, collection and storage of energy with a higher yield and lower energy input than traditional mechano-chemical approaches.

## Recycling of electronics, plastics and other waste streams

Currently, most efforts of developing highly efficient systems for loop-closure focus on recycling and upcycling of biological waste (e.g., food, black/grey/yellow water)^79^, while solutions for synthetic waste (e.g., electronic waste, plastics, consumables) are largely unexplored, and its current management is unviable in long-term missions^26^.

Valuable metals (Fe, Cu, rare earth elements, Al, Si, Zn), including precious metals (Au, Pt and platinum group elements), as well as certain non-metals (Cl, P, N and even O) can be recovered from metallic structures and electronic devices with biology^80^. Reclaiming metals from electronic waste (e.g., computer components, switchboards, solar panels) could reduce the need for resupply and/or the more effortful sourcing from in situ resources through e.g., biomining (see section 'Biomining')^50,54^. Physicochemical processes are available; however, bioleaching technologies are considered more environmentally friendly and sustainable, in terms of costs and energy requirements^81^. The biochemical reactions implicated in these processes are analogous to those involved in biomining (see the section 'Biomining'), hence similar microorganisms and biotechnologies could be used to recycle electronic waste^82^.

Plastics have become indispensable in our everyday life on Earth being used, for instance, in the construction, packaging and manufacturing industries. Beyond the common Earth-analogue applications, plastics, particularly those with high strength and durability, play a key role in supporting human activities in space, as components of spacecraft and spacesuits^16^. Most plastics are composed of organic polymers derived from non-renewable fossil fuels^83^. In an environment such as space, where fossil fuels are not available, recycling and upcycling of plastics will be important to (i) obtain feedstock for manufacturing to produce new consumables, (ii) re-use and recycle resources and thus close the loop on carbon-based feedstocks, and (iii) reduce waste disposal. Microorganisms have been shown to break down microplastics into metabolisable compounds that can support growth. The process, called biodegradation, offers exciting paths towards a circular bioeconomy^83^, and researchers at the UK Centre for Astrobiology (University of Edinburgh, UK) are exploring if these microbial processes could also be leveraged for waste recycling in space. Modern synthetic biology approaches could be used to programme microbial pathways/functions tailored to extra-terrestrial environments^43,48^, and use plastics as feedstock for upcycling and produce useful molecules^84,85^. Complementary to the microbial plastic biodegradation stands microbial production of plastics (e.g., bioplastics). Certain microorganisms can use a variety of feedstock, including CO_2_, CH_4_, or waste biomass, to produce bioplastics such as polyhydroxyalkanoates^4^. These processes will enhance sustainability by means of loop-closure, also because bioplastics are more readily biodegradable than fossil-fuel-based polymers.

While not direct functions of life-support (as is the production of e.g., food, oxygen, water), all the foregone microbial processes can support LSS and vice versa, making long-duration spaceflight more sustainable. For instance, metals deriving from electronic waste could feed LSS compartments containing plants or microorganisms, which in turn can enhance processes directly linked to LSS (Fig. 1)^50^.

## Soil remediation

Heavy metals and toxic compounds, such as perchlorates, could be removed from the Lunar and Martian regolith, allowing it to be used in soil formation for food production^86^. To this aim, the removal of toxic compounds is necessary due to the potential risk that their accumulation in plant tissues poses for the crew^87^. As described above (see the section 'Biomining'), microorganisms can bind and mobilise specific elements from Lunar and Martian regolith^53,88^. This, together with the removal of toxic elements and compounds (bioremediation), can improve the quality of regolith as a plant-growth substrate^89^, drastically reducing the required resources for the continued operation of plant-based LSS. Ultimately, this circular approach combines key elements of the processes described so far while enabling the production of the food needed to sustain humans off-Earth and minimizing the resources that need to be transported from Earth.

Bioremediation may be supported with proteobacteria, e.g., *Sphingomonas*, as well as fungi, such as *Penicillium* spp.^90,91^. Genetic engineering of these organisms or other species could enable the removal of perchlorates (e.g., conversion of perchlorates into molecular chloride and oxygen^87^), as well as heavy metals, radioactive species accumulation and conversion^92^, acids, salts, and organic pollutants from extra-terrestrial regolith.

As microorganisms catalyse very specific reactions, their use can result in highly efficient use of resources, while leaving non-toxic compounds unaltered. In addition, the energy requirements for bioremediation are generally lower than physicochemical alternatives such as heat-treatment to decompose perchlorates. The last point might be offset by required nutrients, which are not guaranteed to be available in situ. However, these could be derived from the other biological LSS compartments, including those described above. Current knowledge on the applicability of these processes in space is limited, therefore a better understanding of the mechanisms of bioremediation, microbial behaviour in the space environment and its potential for soil formation is desirable.

## Technology needs and future research

A common technological denominator across most of the applications discussed thus far is the need to provide a controlled environment for the microbes and the functions they support or enable. Microbial processes depend on temperature, pressure, oxygen availability (or absence), pH, gravitational and radiation conditions, and other factors^93,94^. These aspects drive (i) the technological need for bioreactors tailored to providing the appropriate environment for specific processes, and (ii) the research needed to understand the effects of the space environment on these processes, to identify the conditions that maximise yield while minimising resources, engineering, and operational requirements.

From a technological perspective, the importance of developing space-ready bioreactors is paramount. These will likely need to be specialised for the application they support, although commonalities among them are expected, which include: (i) providing and maintaining a controlled environment (temperature, pressure, liquid/gas composition), (ii) the ability for data collection in terms of the viability of the engineered system and the microbes therein, as well as its performance (including the chemical and physical status of the bulk medium and gas as applicable), (iii) the presence of interfaces to provide precursors, receive products, and attach downstream processing equipment, (iv) the ability for on-demand sample collection, (v) the ability to operate autonomously, at least partially; and (vi) the appropriate level of containment to adhere to planetary protection guidelines, which is particularly relevant for future Martian settlements (notably, closed-loop systems have been identified as a potential solution from this perspective)^95^.

There will be differences depending on the application, for example, the surface area to volume ratio (SAVR), e.g., microbiologically supported direct air-capture of CO_2_ with photoautotrophs requires high SAVR to maximise the molar ratio of CO_2_ to microbes and cellular exposure to light^96^. In contrast, some pharmaceutical synthesis processes may be performed anaerobically and hence may not depend on SAVR^39^. Another aspect that may vary in culture volume: applications requiring high-SAVR will likely generate design drivers with serpentine-like culturing volumes, particularly for phototrophic organisms (similar to high-performance heat-exchangers), while low-SAVR applications may be enabled with simpler cylindrical tanks. This, in turn, may drive the systems’ need for fluid flow and mixing^94^. There are multiple other aspects to consider that can also impact process efficacy, which is especially applicable in bioreactors without stirring. Namely, these refer to mass transport phenomena, both intra- and inter-phase (liquid–solid, gas–liquid, gas–solid), which are dependent upon concentration gradients, temperature, etc., and must be considered during bioreactor design.

Two technological needs relevant across applications will be data acquisition and sample collection, which in several cases will require gas/liquid/solid separation. On the Lunar and Martian surfaces, this may occur naturally owing to the presence of (partial) gravity. Operations taking place in microgravity (e.g., space stations and Mars-transit vehicles), however, pose a more challenging engineering problem. Of particular interest from a biological perspective would be the development of automated and real-time monitoring systems (growth and production rate, titre and yield, pH, pO_2_, as well as inputs and outputs), to allow characterization of system performance.

A requirement for any microbial-based biotechnology is liquid water^97^, one of the most critical commodities for any long-term space activity. On the ISS, water is currently obtained by the Water Recovery System, which is part of ECLSS^18^: urine, humidity condensate, and human waste are recycled to generate potable water, through multiple processes including distillation, filtration, high-temperature catalysis and chemical disinfection^98^. This may not be sufficient on long-duration missions and, ultimately, settlements. Therefore, water recovery must be improved by a more complete loop-closure (Fig. 1). Moreover, additional water could be obtained from different locations on the Moon and Mars, or from asteroids, by mechanic/physical processes and/or biomining (see the section 'Biomining')^50^.

Additional technology needs include the compartmentalisation of applications requiring a certain level of containment and isolation from others. Some applications like MICP, soil-bioremediation and myco-architecture may require exclusively dedicated compartments to protect the rest of the surface base from otherwise unwanted microbial communities and the regolith, which can have detrimental effects on crew health and equipment, while others may be performed in the same habitat-section (Fig. 1). Space conditions, especially the high levels of radiation, showed to induce mutations, changing the genotype of microbial systems in a way that the phenotype was impacted^93^. These could affect the reliability and robustness of microbial processes in space. Hence, technologies should include radiation shielding where appropriate, strict and frequent control of phenotypes and genotypes, as well as redundancies (e.g., protected storage of the primary inoculum as backup).

**Fig. 2:**
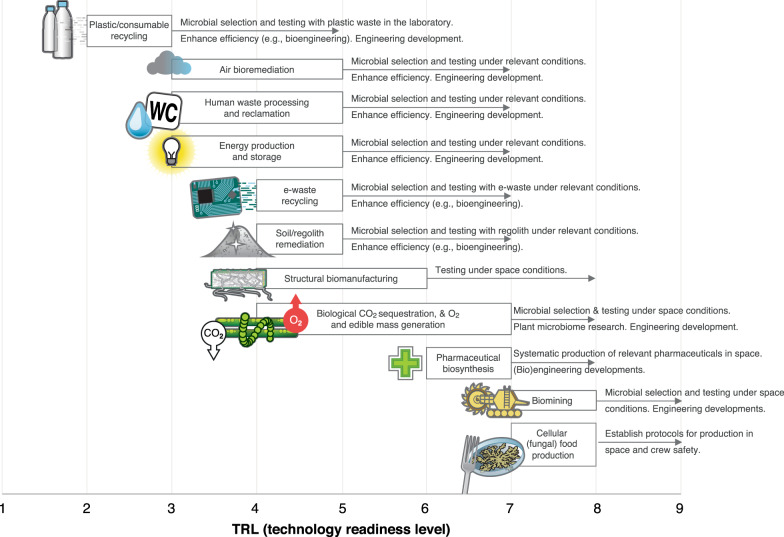
Schematic representation of current technology readiness level (TRL) for the considered biotechnologies. Arrows indicate the specific research and development needed to advance the TRL of each technology. TRL is defined as indicated by NASA.

Each of the categories described in the section 'Habitat air bioremediation' requires research to increase their TRL to nine, as described in Fig. 2. This research will, in turn, need dedicated platforms to perform research, for instance, access to microgravity and partial gravities^40,99^, as well as space radiation. Research needs, in addition to what is described in the former section, include biofilm control strategies. Biofilm formation has been a problem in every space station from Salyut 6 to ISS and can cause operational disruption of ECLSS equipment. This has been addressed via resupply from Earth, which will not be possible for long-term missions beyond LEO^100^. Engineered systems hosting microbial cultures for desired applications will be naturally exposed to the risk of unwanted microbial biofilm formation (i.e., fouling).

This discussion motivates the following set of open research questions: (i) how can microbial biotechnology enhance the sustainability of long-term deep-space exploration missions and settlements, waste-recycling, while preserving extra-terrestrial environments? (ii) How can these processes be conducted while adhering to planetary protection guidelines? (iii) What technologies are needed to monitor process efficiency? For each application: (iv) what microorganisms (including genetically modified) and cultivation conditions optimise the process in the relevant gravitational and radiation environment? (v) What are the bioreactor, operational, and infrastructure requirements applicable to implementing the process? (vi) How do we collect, extract, and refine aliquots and final products obtained from the processes?

## Benefits for Earth

Space exploration has always been a catalyst and testbed for the development of new technologies with applications on Earth (e.g., spin-off technologies)^101^. Space science’s implications for solving terrestrial environmental issues are not new, either. In fact, space activities are recognised by the United Nations as “essential tools for realising the achievement of the SDGs”^11,12^. Further, space-based technologies are indispensable for monitoring weather and vegetation patterns (natural as well as anthropogenic), deforestation, water resources, plastic pollution, carbon emissions and climate change. More specifically, space biotechnologies listed in this work could provide powerful tools to support a number of specific SDGs, including ‘Zero Hunger’ (Goal 2), ‘Good Health and Well-Being’ (Goal 3), ‘Clean Water and Sanitation’ (Goal 6), ‘Affordable and Clean Energy’ (Goal 7), ‘Industry, Innovation and Infrastructure’ (Goal 9), ‘Sustainable Cities and Communities’ (Goal 11), ‘Responsible Consumption and Production’ (Goal 12), and ‘Climate Action’ (Goal 13)^11,12^. In the future, terrestrial environmental issues may be addressed by using space biotechnologies deliberately designed to enhance sustainability: systems that allow tight loop-closure and near-ideal circular operation of resources on a small scale are exemplary for application at a global scale. This kind of tangible public benefit is key not only for justifying space exploration to funding agencies and governments but for motivating scientists to invest their time and energy in space exploration at a time when multiple overlapping crises threaten the stability of our civilization and its ecological support systems.

Space agencies and organizations are aware of the significant potential of this concept. An increasing number of calls for proposals are being launched with the aim of developing strategies to tackle environmental problems on Earth by using space resources. In 2021, ESA (i.e., ‘ESA Circular Economy Kick-Start Competition’)^102^ and NASA (i.e., ‘Orbital Alchemy Challenge’)^103^ launched relevant competitions. In 2022, the Center for the Advancement of Science in Space (CASIS) announced the ‘Beyond Plastics’ sustainability challenge (i.e., ‘CASIS Beyond Plastics’)^104^, with the motto “What if the next great discovery to improve our planet came from research off the planet?”. Notably, two out of the three finalist projects, including the winner, involved the use of microorganisms.

Many of the microbial-based space technologies and strategies presented here have the potential to be transferred and pose benefits to terrestrial applications. Soil remediation techniques developed for application on other terrestrial bodies could be used to help react to soil pollution on Earth, identifying or engineering organisms suitable for cleaning up and degrading specific contaminants, as well as desertification, using desiccation-resistant organisms. Similarly, technologies or plant/microbial genetic engineering could be used to allow for crops to grow in less space with limited resources and deliver comparable or higher yields.

Increased bacterial virulence in space flight has the potential to help identifying new targets for combating drug resistance^37^. On-demand space manufacturing of pharmaceuticals could result in simpler (e.g., fewer steps) yet flexible drug production and purification than on Earth. This may transform the complex and hardly sustainable production processes currently in use, replacing them with biomanufacturing and reducing the cost of pharmaceuticals, thus enabling access to critical medication in remote locations with scarce resources. Carbon-capture and -sequestration technologies will have direct transfer potential for Earth application in the effort to counteract climate change.

In the energy transition, hydrogen is considered a cornerstone for seasonal storage, industrial processes, and general grid balancing. Biologically production of hydrogen, utilised in the context of space technology, could advance the discovery of production pathways for green hydrogen on Earth^75^. Research in bio-concrete synthesis and myco-architecture will enhance the ways how microorganisms can be employed for the construction and maintenance of infrastructure. Like space, Earth also has various extreme conditions. Engineering microbes for infrastructure maintenance can solve problems in remote and austere locations. Improved and highly efficient waste recycling, including but not limited to organic/biological, plastic/consumable, and electronic waste, for space applications could be translated back for Earth applications to tackle terrestrial waste management issues, landfill overflow and environmental pollution.

## Conclusions

With this work, we aimed to advocate the urgency of sustainable approaches for human space exploration, and the essential role microbial biotechnologies could play in this. It has been discussed how microbial biotechnologies may support several processes that, in turn, can help decrease supply chain dependency (from Earth) on long-duration deep-space exploration missions to eventually enable human footholds and settlements across the solar system. In parallel, several of these microbially-supported processes may also help close the loop on LSS and other systems, which can preserve resources and extra-terrestrial environments. These two aspects, independently and together, enable space exploration in a sustainable fashion.

To provide appropriate conditions for the maturation of named biotechnologies and to open the field to a wider community, research and innovation are needed. Preliminary studies should be supported in laboratories on Earth, simulation platforms, and in LEO, and full investigations in *cis*-Lunar space and on the Lunar surface. Augmenting existing space-based facilities to increase the capacity for space experiments, in terms of sample replicates and experimental conditions, should also be considered to improve the impact of the preliminary studies. Funding should take into account the high cost (and time) of genetic engineering and synthetic biology, on which these technologies will likely heavily rely.

Finally, the potential of these technologies for terrestrial benefits has been discussed, with specific reference to the United Nation SDGs. If ultra-efficient approaches to sustaining human life and economic activity off-Earth can be adapted towards meaningfully solving problems on our planet, space exploration will have yielded a manifold return on public investments and will thereby become more sustainable not only technologically, but also politically. The debate about the public benefits of space travel and exploration is healthy and necessary, but in the field of sustainability, space advocates may now have an opportunity to win this debate for a generation.

## References

[1] Secure World Foundation. *Space Sustainability—A Practical Guide* (Secure World Foundation, 2018).

[2] Williamson RA (2012). Assuring the sustainability of space activities. Space Policy.

[3] Newman CJ, Williamson M (2018). Space sustainability: reframing the debate. Space Policy.

[4] Berliner AJ (2021). Towards a biomanufactory on Mars. Front. Astron. Space Sci..

[5] Gòdia F (2002). MELISSA: a loop of interconnected bioreactors to develop life support in Space. J. Biotechnol..

[6] Keller, R. J. et al. Biologically-based and physiochemical life support and in situ resource utilization for exploration of the solar system—reviewing the current state and defining future development needs. *Life* **11**, 844 1–41 (2021). 10.3390/life11080844 PMC8398003 34440588

[7] Nangle SN (2020). The case for biotech on Mars. Nat. Biotechnol..

[8] Lawrence A (2022). The case for space environmentalism. Nat. Astron..

[9] Verseux C (2016). Sustainable life support on Mars—the potential roles of cyanobacteria. Int. J. Astrobiol..

[10] Matthews JJ, McMahon S (2018). Exogeoconservation: protecting geological heritage on celestial bodies. Acta Astronaut..

[11] United Nations Office for Outer Space Affairs. *Guidelines for the Long-term Sustainability of Outer Space Activities Conference Room Paper by the Chair of the Working Group on the Long-term Sustainability of Outer Space Activities I. Context of the Guide*, vol. V.18-04441 1–20 (Committee on the Peaceful Uses of Outer Space, 2018). **This document highlights the roles of space activities in realizing the achievement of the United Nations Sustainable Development Goals**.

[12] United Nations. *The 17 Goals|Sustainable Development* https://sdgs.un.org/goals (2023)

[13] Montague M (2012). The role of synthetic biology for in situ resource utilization (ISRU). Astrobiology.

[14] Castelein SM (2021). Iron can be microbially extracted from Lunar and Martian regolith simulants and 3D printed into tough structural materials. PLoS ONE.

[15] Berliner AJ (2022). Space bioprocess engineering on the horizon. Commun. Eng..

[16] Averesch, N. J. H. et al. Biomanufacturing for space-exploration—what to take and when to make. *Preprints.Org* 1–13 10.20944/preprints202207.0329.v1 (2022). **A brilliant outline of strategic approaches to integrate biomanufacturing into space missions**.

[17] Nickerson CA, Medina-Colorado AA, Barrila J, Poste G, Ott CM (2022). A vision for spaceflight microbiology to enable human health and habitat sustainability. Nat. Microbiol..

[18] Averesch NJH (2021). Choice of microbial system for in-situ resource utilization on Mars. Front. Astron. Space Sci..

[19] Dallbauman LA, Finn JE (1999). Adsorption processes in spacecraft environmental control and life support systems. Stud. Surf. Sci. Catal..

[20] Vinayak, V. Chapter 20 - Algae as sustainable food in space missions. In *Biomass, Biofuels, Biochemicals: Circular Bioeconomy: Technologies for Biofuels and Biochemicals* (Eds. Varjani, S., Pandey, A., Bhaskar, T., Mohan, S. V. & Tsang, D. C. W.) 517–540 (Elsevier, 2022).

[21] International Energy Agency, I. *Direct Air Capture A Key Technology for Net Zero*, Vol. 76 (International Energy Agency, 2022).

[22] Hu G, Li Y, Ye C, Liu L, Chen X (2019). Engineering microorganisms for enhanced CO_2_ sequestration. Trends Biotechnol..

[23] Voskian S, Hatton TA (2019). Faradaic electro-swing reactive adsorption for CO_2_ capture. Energy Environ. Sci..

[24] Mapstone LJ, Leite MN, Purton S, Crawford IA, Dartnell L (2022). Cyanobacteria and microalgae in supporting human habitation on Mars. Biotechnol. Adv..

[25] Detrell G (2021). *Chlorella vulgaris* photobioreactor for oxygen and food production on a Moon base—potential and challenges. Front. Astron. Space Sci..

[26] Linne, D. L. et al. Waste management options for long-duration space missions: when to reject, reuse, or recycle. In *Proc. American Institute of Aeronautics and Astronautics 7th Symposium on Space Resource Utilization* (Eds. Balasubramaniam, R. & Hegde, U. G.) 1–9 (AIAA SciTech, 2014).

[27] Schneider, W. F. et al. NASA environmental control and life support technology development and maturation for exploration: 2019 to 2020 overview. In *Proc. 2020 International Conference on Environmental Systems* 1–12 (ICES 2020).

[28] Steinberg LM, Kronyak RE, House CH (2017). Coupling of anaerobic waste treatment to produce protein- and lipid-rich bacterial biomass. Life Sci. Space Res..

[29] Yadav AN, Kour D, Ahluwalia AS (2021). Soil and phytomicrobiomes for plant growth and soil fertility. Plant Sci. Today.

[30] Santos LF, Olivares FL (2021). Plant microbiome structure and benefits for sustainable agriculture. Curr. Plant Biol..

[31] Langenfeld NJ (2021). Optimizing nitrogen fixation and recycling for food production in regenerative life support systems. Front. Astron. Space Sci..

[32] Woo SL, Pepe O (2018). Microbial consortia: promising probiotics as plant biostimulants for sustainable agriculture. Front. Plant Sci..

[33] Mitter EK, Tosi M, Obregón D, Dunfield KE, Germida JJ (2021). Rethinking crop nutrition in times of modern microbiology: innovative biofertilizer technologies. Front. Sustain. Food Syst..

[34] Porto de Souza Vandenberghe L (2017). Potential applications of plant probiotic microorganisms in agriculture and forestry. AIMS Microbiol..

[35] Naseer, I. et al. *Biofertilizers for Sustainable Agriculture and Environment* Vol. 55 (Agrobios Indian Publications, 2019).

[36] Monje O (2020). Hardware validation of the advanced plant habitat on ISS: canopy photosynthesis in reduced gravity. Front. Plant Sci..

[37] Zea, L. Drug discovery and development in space. In *Proc. 66th International Astronautical Congress, IAC-15-A1.8.1,x27627* 273–282 (IAF, 2015).

[38] Tanaka H (2011). Improvement in the quality of hematopoietic prostaglandin D synthase crystals in a microgravity environment. J. Synchrotron Radiat..

[39] Pathak, Y. V., Araujo dos Santos, M. & Zea, L. *Handbook of Space Pharmaceuticals* (Springer, 2020).

[40] Nickerson, C. A., Pellis, N. R. & Ott, C. M. *Effect of Spaceflight on Human and Analogue Culture and Spaceflight Microbial Cells* (Springer, 2016).

[41] Amselem S (2019). Remote controlled autonomous microgravity lab platforms for drug research in space. Pharm. Res..

[42] Du B (2011). Evaluation of physical and chemical changes in pharmaceuticals flown on space missions. AAPS J..

[43] McNulty MJ (2021). Evaluating the cost of pharmaceutical purification for a long-duration space exploration medical foundry. Front. Microbiol..

[44] Menezes, A. A., Cumbers, J., Hogan, J. A. & Arkin, A. P. Towards synthetic biological approaches to resource utilization on space missions. *J. R. Soc. Interface* **12**, 20140715. -20 (2014). **This paper reviews highlights the potential for synthetic biology in human space exploration and ISRU**. 10.1098/rsif.2014.0715 PMC4277073 25376875

[45] Chen X, Li C, Liu H (2021). Enhanced recombinant protein production under special environmental stress. Front. Microbiol..

[46] Turroni S (2020). Gut microbiome and space travelers’ health: state of the art and possible pro/prebiotic strategies for long-term space missions. Front. Physiol..

[47] Voorhies AA (2019). Study of the impact of long-duration space missions at the International Space Station on the astronaut microbiome. Sci. Rep..

[48] De Roy K, Marzorati M, Van den Abbeele P, Van de Wiele T, Boon N (2014). Synthetic microbial ecosystems: an exciting tool to understand and apply microbial communities. Environ. Microbiol..

[49] Menezes, A. A., Montague, M. G., Cumbers, J., Hogan, J. A. & Arkin, A. P. Grand challenges in space synthetic biology. *J. R. Soc. Interface* **12**, 20150803 1–7 (2015). 10.1098/rsif.2015.0803 PMC4707852 26631337

[50] Santomartino, R., Zea, L. & Cockell, C. S. The smallest space miners: principles of space biomining. *Extremophiles* **26**, 7 1–19 (2022). 10.1007/s00792-021-01253-w PMC8739323 34993644

[51] Gumulya Y, Zea L, Kaksonen AH (2022). In situ resource utilisation: the potential for space biomining. Miner. Eng..

[52] Schippers A (2013). Biomining: metal recovery from ores with microorganisms. Geobiotechnology. I. Adv. Biochem. Eng..

[53] Volger R (2020). Mining Moon & Mars with microbes: biological approaches to extract iron from Lunar and Martian regolith. Planet. Space Sci..

[54] Cockell, C. S. & Santomartino, R. Mining and microbiology for the solar system silicate and basalt economy. In *Space Manufacturing Resources: Earth and Planetary Exploration Applications* (eds Hessel, V., Stoudemire, J., Miyamoto, H. & Fisk, I. D.) 163–185 (Wiley, 2022).

[55] Cockell CS (2021). Microbially-enhanced vanadium mining and bioremediation under micro- and Mars gravity on the International Space Station. Front. Microbiol..

[56] Cockell CS (2020). Space station biomining experiment demonstrates rare earth element extraction in microgravity and Mars gravity. Nat. Commun..

[57] Roberto FF, Schippers A (2022). Progress in bioleaching: part B, applications of microbial processes by the minerals industries. Appl. Microbiol. Biotechnol..

[58] Franz, H. B., King, P. L. & Gaillard, F. Chapter 6 - Sulfur on Mars from the atmosphere to the core. In *Volatiles in the Martian Crust* (eds. Filiberto, J. & Schwenzer, S. P.) 119–183 (Elsevier Inc., 2019).

[59] Santomartino R (2020). No effect of microgravity and simulated Mars gravity on final bacterial cell concentrations on the International Space Station: applications to space bioproduction. Front. Microbiol..

[60] Castro-Alonso MJ (2019). Microbially induced calcium carbonate precipitation (MICP) and its potential in bioconcrete: microbiological and molecular concepts. Front. Mater..

[61] Beruto DT, Barberis F, Botter R (2005). Calcium carbonate binding mechanisms in the setting of calcium and calcium–magnesium putty-limes. J. Cult. Herit..

[62] Okyay TO, Nguyen HN, Castro SL, Rodrigues DF (2016). CO_2_ sequestration by ureolytic microbial consortia through microbially-induced calcite precipitation. Sci. Total Environ..

[63] Kang CH, Han SH, Shin Y, Oh SJ, So JS (2014). Bioremediation of Cd by microbially induced calcite precipitation. Appl. Biochem. Biotechnol..

[64] Huang Z, Jiang D, Lu L, Ren ZJ (2016). Ambient CO_2_ capture and storage in bioelectrochemically mediated wastewater treatment. Bioresour. Technol..

[65] Kahre MA, Murphy JR, Haberle RM (2006). Modelling the Martian dust cycle and surface dust reservoirs with the NASA Ames general circulation model. J. Geophys. Res. Planets.

[66] Dikshit R, Gupta N, Dey A, Viswanathan K, Kumar A (2022). Microbial induced calcite precipitation can consolidate martian and lunar regolith simulants. PLoS ONE.

[67] Liu Y (2008). Control of lunar and martian dust-experimental insights from artificial and natural cyanobacterial and algal crusts in the desert of Inner Mongolia, China. Astrobiology.

[68] Rajasekar, A., Moy, C. K. S. & Wilkinson, S. MICP and advances towards eco-friendly and economical applications. *IOP Conf. Ser. Earth Environ. Sci*. **78**, 012016 (IOP Publishing, 2017).

[69] Ganendra G (2014). Formate oxidation-driven calcium carbonate precipitation by *Methylocystis parvus* OBBP. Appl. Environ. Microbiol..

[70] Attias N (2020). Mycelium bio-composites in industrial design and architecture: comparative review and experimental analysis. J. Clean. Prod..

[71] Rothschild, L. J. et al. *Myco-architecture off planet: Growing Surface Structures at Destination.* NIAC 2018 Phase I Final Report. 2019-08-30T21:35:36+00:00Z (NASA Ames Research Center: Mountain View, CA, USA, 2018).

[72] Shunk, G. K., Gomez, X. R., Kern, C. & Averesch, N. J. H. Growth of the radiotrophic fungus *Cladosporium sphaerospermum* aboard the International Space Station and effects of ionizing radiation. *bioRxiv* 2020.07.16.205534 (2022). 10.3389/fmicb.2022.877625 PMC9294542 35865919

[73] Lovley DR (2006). Microbial energizers: fuel cells that keep on going. Microbe.

[74] Hallenbeck PC, Liu Y (2016). Recent advances in hydrogen production by photosynthetic bacteria. Int. J. Hydrog. Energy.

[75] Dahiya S, Chatterjee S, Sarkar O, Mohan SV (2021). Renewable hydrogen production by dark-fermentation: current status, challenges and perspectives. Bioresour. Technol..

[76] Kracke, F., Deutzmann, J. S., Gu, W. & Spormann, A. M. In situ electrochemical H_2_ production for efficient and stable power-to-gas electromethanogenesis. 6194–6203 10.1039/d0gc01894e (2020).

[77] Barton, L. L., Tomei-Torres, F. A., Xu, H. & Zocco, T. Chapter 7—Nanoparticles formed by microbial metabolism of metals and minerals. In *Nanomicrobiology—Physiological and Environmental Characteristics* (eds Barton, L. L., Bazylinski, D. A. & Xu, H.) 145–176 (Springer, 2014).

[78] Kruyer NS, Realff MJ, Sun W, Genzale CL, Peralta-Yahya P (2021). Designing the bioproduction of Martian rocket resource utilization strategy. Nat. Commun..

[79] Lasseur C, Mergeay M (2021). Current and future ways to closed life support systems: virtual MELiSSA conference, Ghent (B) (3-5/11/2020). A review. Ecol. Eng. Environ. Prot..

[80] Urbina J (2019). A new approach to biomining: bioengineering surfaces for metal recovery from aqueous solutions. Sci. Rep..

[81] Valix, M. Bioleaching of electronic waste: milestones and challenges. In *Current Developments in Biotechnology and Bioengineering: Solid Waste Management* (Eds. Wong, J. W. -C., Tyagi, R. D. & Pandey, A.) 407–442 (Elsevier B.V., 2017).

[82] Srichandan H, Mohapatra RK, Parhi PK, Mishra S (2019). Bioleaching approach for extraction of metal values from secondary solid wastes: a critical review. Hydrometallurgy.

[83] Shah AA, Hasan F, Hameed A, Ahmed S (2008). Biological degradation of plastics: a comprehensive review. Biotechnol. Adv..

[84] Tiso T (2022). The metabolic potential of plastics as biotechnological carbon sources—review and targets for the future. Metab. Eng..

[85] Sadler, J. C. & Wallace, S. Microbial synthesis of vanillin from waste poly(ethylene terephthalate). *Green Chem*. **23**,4661 1–8 (2021). 10.1039/d1gc00931a PMC8256426 34276250

[86] Billi D, Gallego Fernandez B, Fagliarone C, Chiavarini S, Rothschild LJ (2021). Exploiting a perchlorate-tolerant desert cyanobacterium to support bacterial growth for in situ resource utilization on Mars. Int. J. Astrobiol..

[87] Duri LG (2022). The potential for Lunar and Martian Regolith simulants to sustain plant growth: a multidisciplinary overview. Front. Astron. Space Sci..

[88] Lehner, B. et al. Bacterial modification of Lunar And Martian Regolith for plant growth in life support systems. In *Proc. 69th International Astronautical Congress, IAC-18,A1,7,7,x42645* 1–7 (IAF, 2018).

[89] Paul AL, Elardo SM, Ferl R (2022). Plants grown in Apollo lunar regolith present stress-associated transcriptomes that inform prospects for lunar exploration. Commun. Biol..

[90] Leitão AL (2009). Potential of *Penicillium* species in the bioremediation field. Int. J. Environ. Res. Public Health.

[91] Matsumura Y, Akahira-Moriya A, Sasaki-Mori M (2015). Bioremediation of bisphenol-a polluted soil by *Sphingomonas bisphenolicum* ao1 and the microbial community existing in the soil. Biocontrol Sci..

[92] Prakash D, Gabani P, Chandel AK, Ronen Z, Singh OV (2013). Bioremediation: a genuine technology to remediate radionuclides from the environment. Microb. Biotechnol..

[93] Horneck G, Klaus DM, Mancinelli RL (2010). Space microbiology. Microbiol. Mol. Biol. Rev..

[94] Zea L (2022). Experiment verification test of the Artemis I ‘Deep Space Radiation Genomics’ experiment. Acta Astronaut..

[95] NASA Interim Directive. *Subject: Biological Planetary Protection for Human Missions to Mars* 1–6 (NASA Interim Directive, 2020).

[96] Fahrion J, Mastroleo F, Dussap CG, Leys N (2021). Use of photobioreactors in regenerative life support systems for human space exploration. Front. Microbiol..

[97] Cockell CS (2021). Bridging the gap between microbial limits and extremes in space: space microbial biotechnology in the next 15 years. Microb. Biotechnol..

[98] Yang, J. et al. Microbiology of the built environment in spacecraft used for human flight. In *Methods in Microbiology* Vol. 45 (Eds. Gurtler, V. & Trevors, J. T.) 3–26, (Elsevier Ltd., 2018).

[99] Zea, L., Santa Maria, S. R. & Ricco, A. J. CubeSats for microbiology and astrobiology research. In *Cubesat Handbook* (Eds. Cappelleti, C. & Battistini, S.) 147–162 (Elsevier Inc., 2021).

[100] Zea, L. et al. Potential biofilm control strategies for extended spaceflight missions. *Biofilm* **2**, 100026 1–20 (2020). 10.1016/j.bioflm.2020.100026 PMC7798464 33447811

[101] I. S. E. C. G. *Benefits Stemming from Space Exploration* 1–22 (ESA Publication, 2013).

[102] European Space Agency. *Circular Economy|ESA Business Applications* https://business.esa.int/funding/invitation-to-tender/circular-economy (2021)

[103] Schlieder, S. *NASA Orbital Alchemy Challenge|NASA* https://www.nasa.gov/orbital-alchemy-challenge (2022)

[104] ISS National Laboratories. *ISS National Lab Sustainability Challenge: Beyond Plastics* https://www.issnationallab.org/research-on-the-iss/2022sustainabilitychallenge/ (2022)

